# Engineered biological neuronal networks as basic logic operators

**DOI:** 10.3389/fncom.2025.1559936

**Published:** 2025-04-28

**Authors:** Joël Küchler, Katarina Vulić, Haotian Yao, Christian Valmaggia, Stephan J. Ihle, Sean Weaver, János Vörös

**Affiliations:** Laboratory of Biosensors and Bioelectronics (LBB), Institute for Biomedical Engineering, ETH Zurich, Zurich, Switzerland

**Keywords:** neuron-on-a-chip, biocomputation, neural networks, neural circuit, Boolean, microelectrode array (MEA), encoding, hybrid intelligence

## Abstract

We present an *in vitro* neuronal network with controlled topology capable of performing basic Boolean computations, such as NAND and OR. Neurons cultured within polydimethylsiloxane (PDMS) microstructures on high-density microelectrode arrays (HD-MEAs) enable precise interaction through extracellular voltage stimulation and spiking activity recording. The architecture of our system allows for creating non-linear functions with two inputs and one output. Additionally, we analyze various encoding schemes, comparing the limitations of rate coding with the potential advantages of spike-timing-based coding strategies. This work contributes to the advancement of hybrid intelligence and biocomputing by offering insights into neural information encoding and decoding with the potential to create fully biological computational systems.

## 1 Introduction

Information processing and learning in artificial intelligence (AI) systems, while diverse, is well-known and understood. At its core, AI learning involves training algorithms that iteratively adjust internal parameters by minimizing the difference between predicted and actual outputs through techniques like backpropagation and gradient descent. Among other aspects, the learning process in AI systems typically involves the use of various activation functions, such as sigmoid, ReLU, or softmax, which determine the output of each neuron based on its inputs (Fahimirad and Kotamjani, 2018). These activation functions are crucial in enabling the system to learn complex patterns and relationships within the data (Adu et al., 2021; Yu et al., 2020; Lee and Kwon, 2010). One of the key aspects of information processing in AI systems is the use of artificial neural networks (ANNs), which are inspired by the structure and function of the human brain (Fahimirad and Kotamjani, 2018).

On the contrary, the mechanisms by which the human brain encodes, processes, and propagates information are complex and not well understood. It appears that different regions of the brain are specialized for various functions, each exhibiting unique properties in terms of information encoding, processing precision, and memory duration (Lavi et al., 2021; Roy et al., 2017; Headley and Paré, 2017). Humans learn quickly and efficiently. They are able to generalize and thus still outperform AI systems (Seo et al., 2021). To accomplish efficient information transmission (and its storage), neurons in the brain communicate through synapses, which transmit signals from one neuron to another. The process involves the release of neurotransmitters, which cross the synaptic cleft and bind to receptors on the receiving neuron. This can initiate an action potential that propagates along the neuron and triggers the release of neurotransmitters at the next synapse. The biological processes that result in the creation of an action potential (output) upon receiving an input can be described as biological activation functions (Hodgkin and Huxley, 1952). This chain of events allows for the transmission of information across different parts of the brain.

In recent years, there has been an emphasis on leveraging biological mechanisms to improve artificial learning, mainly in efficiency and generalizability. These include the development of neuromorphic hardware (Mead, 1990; Indiveri et al., 2011; Chicca et al., 2014) and the implementation of spiking neural networks (SNNs) (Maass, 1997), which are inspired by biological neural networks (De Venuto et al., 2024). Neuromorphic computing replicates the structure of the human brain and may enable highly efficient computing through parallel computation and adaptive learning (Kim and Lee, 2019). SNNs offer an energy-efficient alternative to traditional ANNs due to their spike-based communication and computation mechanisms (Lee et al., 2020; Rathi and Roy, 2023). Reservoir computing provides another biologically-inspired approach that mimics how neural microcircuits process temporal information, utilizing fixed random connections within a recurrent network to reduce training complexity while maintaining powerful computational capabilities (Jaeger and Haas, 2004; Appeltant et al., 2011; Tanaka et al., 2019). This paradigm can be implemented with neuromorphic hardware (Karki et al., 2024). The main limitation of SNNs and neuromorphic hardware is that, as mentioned before, the biological mechanisms of learning and information processing are not fully understood, which complicates accurate implementation (Kwisthout and Donselaar, 2020; Burr et al., 2016). Important questions relate to identifying the aforementioned biological activation functions and finding proper input/output (I/O) encoding mechanisms.

One perspective on biological behavior in terms of information encoding, transmission, and decoding postulates that neurons operate similarly to communication channels (Ikeda and Manton, 2009). In this view, neurons receive inputs, process information, and then produce, to some extent, noisy outputs, effectively acting as conduits for transmitting and transforming information within the brain. This theory draws on the analogy to information theory, where communication channels transmit data between a sender and a receiver (Abramson, 1990; Hamming, 1990; Garliauskas, 2007).

Several coding theories intend to explain how neurons represent and communicate information. Key frameworks include rate coding and temporal coding, such as phase coding, inter-spike interval (ISI) coding, and time-to-first spike (TTFS) coding. Rate coding represents information by the frequency of spikes within a specific time frame. Research suggests that the cortex mainly uses rate coding, as it is resistant to noise due to the averaging effect over time (London et al., 2010). This makes rate coding particularly effective for reliable information transfer, even in highly variable environments. Rate coding carries an implication of temporal averaging which can work well when the stimulus is constant, slowly varying, or does not require a fast reaction. Outside these assumptions, it can be limiting (Gerstner et al., 2014).

Phase coding uses the timing of spikes relative to a periodic signal, which helps capture temporal patterns in sensory information (Kayser et al., 2009). This approach has been shown to strengthen the stability of information carried by spatial and temporal spike patterns, improving the accuracy of sensory representations (Kayser et al., 2009).

Similarly, TTFS coding, which focuses only on the timing of the first spike in response to a stimulus, is valued for its speed in transmitting information, especially in fast sensory environments (Guo et al., 2021). Precise spike timing in TTFS coding plays a key role in interpreting sensory signals (Guo et al., 2021; Park et al., 2019).

ISIs are important in neural coding, as they provide information on the statistical properties of stimuli. Neurons can adapt to different signal features by adjusting their firing intervals (Lundstrom and Fairhall, 2006). ISIs can reflect stimulus variability, highlighting the role of spike timing patterns in sensory processing (Lundstrom and Fairhall, 2006). Analyzing ISI variability also provides insights into neural behavior and the characteristics of incoming input signals (Kuebler and Thivierge, 2014). This is particularly important in cases where changes in neural behavior need to be quantified, such as during neuroplasticity, i.e., the learning processes of neurons.

The encoding mechanisms and activation processes of biological neurons must be further explored in neural systems, particularly in the context of developing biologically inspired AI systems.

While numerous *in silico* studies have explored I/O transformations across various complexity levels of neural systems (Zador et al., 1991; Sidiropoulou et al., 2006; Cazé et al., 2013; Beniaguev et al., 2021; Ünal and Ba s cift ci, 2023), studying and observing these phenomena in living biological systems remain considerably more limited and challenging (Lampl and Yarom, 1997; Zeng et al., 2019; Kagan et al., 2022). Since currently the brain as a whole represents an incomprehensible complex system, it is helpful, if not essential to break down the complexity to understand the processes of communication and information transmission. One way to obtain well-defined and reproducible biological neural systems suitable for studying such phenomena is to form engineered *in vitro* neuronal networks with controlled topology and defined directionality and connectivity (Courte et al., 2018; Forró et al., 2018; Maoz, 2021). Additionally, it is necessary to interact with the system in order to provide controlled inputs and to read out the corresponding outputs. One way to achieve network directionality and feed-forward information flow is to culture neurons inside custom-designed polydimethylsiloxane (PDMS) microstructures and to place them on top of (high-density) microelectrode arrays (HD-MEAs). This allows for studying I/O transformations of simplified neural systems by allowing both the observation of the system through the extracellular recording and the manipulation of the system through extracellular voltage stimulation (Bakkum et al., 2013; Shahaf and Marom, 2001; Duru et al., 2022; Ihle et al., 2022; Kagan et al., 2022). This methodology has recently been applied to leverage biological systems for various forms of problem-solving (Zeng et al., 2019; Kagan et al., 2022; Smirnova et al., 2023).

To accomplish efficient hybrid computation, three key components are essential: First, we need to define and understand the basic computation in the biohybrid system, identifying the fundamental processing units and their operational principles. Second, we need to be able to use those basic computational elements as building blocks to create more complex systems with hierarchical organization. Third, precise control over how these elements communicate is needed, specifically controlling the number, strength, and direction of synaptic connections to ensure predictable information flow. Recent works by Kagan et al. and Smirnova et al., while demonstrating the computational capabilities of biological neural systems, lack these key components of control and understanding required for efficient hybrid computation. In our previous work, we aimed to achieve control over the location of synaptic connections (Mateus et al., 2022) and to understand the information propagation depending on the size of the transmission cable (axon bundles) (Amos et al., 2024; Vulić et al., 2024), in this work we address the first requirement mentioned above by characterizing the basic computational capabilities of a simplified biological neural network. We present a feedforward biological neural network with a two-input-one-output topology. The topology is controlled by confining neuronal growth within PDMS microstructures. We interact with a network through high-density microelectrode arrays. We encode external inputs using specific stimulation patterns and represent output information through different encoding schemes. First, we explore the limitations of information transmission in our system through simple encoding schemes. We identify the optimal encoding strategy and valorize the network's reproducible behavior to create non-linear biological transfer functions and perform fundamental logic operations. Characterizing these concepts within biological systems could provide valuable insights into the validity and relevance of the principles currently applied in bio-inspired computing.

## 2 Materials and methods

### 2.1 PDMS microstructures

**Figure 1:**
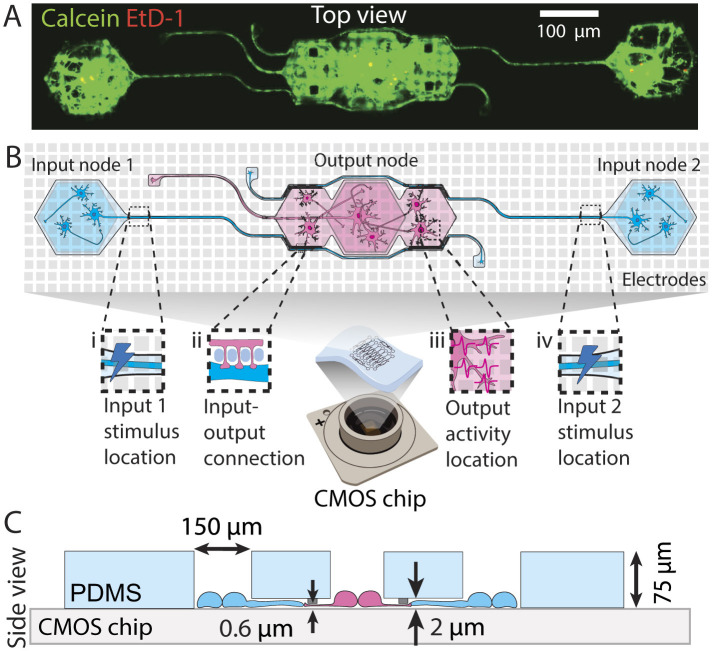
Engineered feedforward neuronal networks on a chip. **(A)** Microscopy example image of a neuronal network defined by a PDMS microstructure. The network consists of two input nodes with microchannels guiding the input axons toward the output node. **(B)** Top view schematic of a network describing (i) the location of the external stimulus applied at the beginning of the first input axon channel, (ii) the putative synaptic connection formed between the input axon and output dendrites through submicron tunnels, (iii) part of the area where output action potentials are recorded from, and (iv) the location of the external stimulus applied at the beginning of the second input axon channel. **(C)** Cross-section schematic of the same microstructure showing the microchannel and submicron tunnel heights, well opening sizes, and the total PDMS thickness.

PDMS microstructures for cell and axon guidance were designed using CAD software (AutoCAD 2021) and fabricated by Wunderlichips (Zurich, Switzerland). The fabrication process is elaborated in previous publications (Mateus et al., 2022; Vulić et al., 2024). In this study, all microstructures feature wells for cell seeding, microchannels impermeable to soma but accessible to neurites, and submicron tunnels designed to prevent axonal outgrowth while allowing penetration of dendritic spines to assure the directionality of action potential propagation. Schematics illustrating these microstructures are presented in Figure 1. The full thickness of the PDMS microstructure measures 75 μm, with 2 μm high microchannels, and a submicron tunnel area with a height of 600 nm. The microstructure was designed to engineer feed-forward neuronal networks consisting of two presynaptic (input) nodes connecting to the postsynaptic (output) node. These networks will from now on be referred to as 2-in-1-out networks. Directionality is achieved by using 250 nm-wide PDMS tunnels (see Figure 1B iii) that restrict the input axon from growing through, while still allowing dendritic spines from output neurons to penetrate and form synaptic connections with the input (for more details, see previous work Mateus et al., 2022).

### 2.2 Microelectrode arrays

Microelectrode arrays (MEAs) used in the experiments are high-density complementary metal-oxide-semiconductor (HD-CMOS) MEAs. The small MaxOne HD-MEA chips with flat surface topology (Maxwell Biosystems, Switzerland) consist of 26,400 electrodes distributed on a 3.85 × 2.10 mm^2^ surface with an electrode pitch of 17.5 μm. It is possible to record from 1,020 electrodes simultaneously. These electrodes can be chosen arbitrarily and are routed to the amplifiers through a switch matrix. The data is sampled at 20 kHz. Extracellular stimulation is possible on the same electrodes and is performed with 32 independent stimulation buffers.

### 2.3 Substrate preparation

The preparation begins with the pre-cleaning of the reused MEA, which can be omitted if the new sterile chip is used. Cleaning involves leaving a chip in 4% Tergazyme (1304-1, Alconox) overnight, rinsing with ultrapure water (Milli-Q, Merck-MilliPore), and then leaving the chips for circa 30 min in 70% ethanol solution. This is followed by three rinses with ultrapure water. Subsequently, the MEA surface is dried as much as possible using a nitrogen gun. Next, the chip is functionalized with Poly-D-Lysine (PDL) (P6407, Sigma Aldrich). The solution is 0.1 mg/mL of PDL in phosphate-buffered saline (PBS) (10010-023, ThermoFisher). 100 μL of the PDL solution is pipetted onto the chip surface, and the chip is left for a minimum of 45 min before the chip is rinsed three times with Milli-Q water to remove unbound PDL polymers. The chip is then dried with a nitrogen gun. PDMS microstructure is then placed on the chip surface with tweezers, ensuring that it is as straight as possible and avoiding contact of the tweezers with the chip surface. Once positioned, the microstructure is gently pressed flat onto the chip. Subsequently, 1 mL of warm PBS is added, and the chip is desiccated for a minimum of 5 min or until no bubbles are observed on the surface of the microstructure.

### 2.4 Visualization of PDMS microstructure with a CMOS MEA

We employ a custom Python script that generates a voltage map showing the location of electrodes beneath the PDMS microstructure. The example of a voltage map for this PDMS microstructure can be seen in Supplementary Figure S1. This enables us to locate and select the electrodes covering individual networks within the PDMS microstructure. The method is described in detail in our previous publication (Duru et al., 2022).

### 2.5 Cell culture

The cells were cultured in NeuroBasal medium (NB) (21203-049, ThermoFisher) (Brewer et al., 1993). Fresh NB complete medium was prepared, consisting of a 2% solution of B-27 supplement (17504-044), a 1% solution of Penicillin-Streptomycin (P-S) (15070-063), and a 1% solution of GlutaMAX (35050-061), all sourced from ThermoFisher.

#### 2.5.1 Cell dissociation

Primary hippocampal neurons were obtained from E18 embryos of pregnant Sprague-Dawley rats (EPIC, ETH Phenomics Center) for use in the experiments. All animal procedures were approved by the Cantonal Veterinary Office Zurich. The embryonic neuronal tissue was dissected and stored in Hibernate E medium (Thermo PN) on ice. Cell dissociation commenced by digesting the tissue in a solution containing 50 mg/mL Bovine Serum Albumin (BSA) (A7906, Sigma-Aldrich), 1.8 mg/mL D-glucose (Y0001745, Sigma-Aldrich), and 0.5 mg/mL papain (P5306, Sigma-Aldrich) dissolved in sterile PBS. Prior to dissociation, the solution was warmed to 37°C, filtered through a 0.2 μm filter, and supplemented with 1 mg/mL DNAse (D5025, Sigma-Aldrich). The tissue was incubated in the papain solution for 15 minutes at 37°C, followed by replacement with NB medium containing 10% fetal bovine serum (10500056, ThermoFisher) to halt the digestion process. Subsequently, two washes with NB medium were performed, with a 5-min interval between each wash. Trituration was then carried out, followed by cell counting using the Cell Countess system (Invitrogen). Cells dissociated from a single pregnant rat were considered as one biological replicate.

#### 2.5.2 Cell seeding and maintenance

Prior to cell seeding onto the chip substrate, PBS was gradually replaced with NB complete medium, avoiding the reformation of air bubbles in the microchannels. Cells were seeded in suspension with a concentration of 70,000 cells per MEA (which corresponds to roughly 250 cells/mm^2^). MEA chips were then transferred into the incubator at 37 °C, 90% humidity, and 5% CO_2_. 20–30 min upon seeding, cells were re-suspended in the chip by pipetting up and down to increase the probability of more cells falling into the PDMS wells. The first medium exchange was done one day after seeding to remove the dead cells and cell debris and the medium was then exchanged twice per week.

### 2.6 Experimental setup

The experimental setup involved stimulation and recording of the neurons topologically constrained to form 2 input node and 1 output node networks (see Figure 1A). Each chip contained up to 10 independent networks The experiments were performed in the third week of culture to ensure the functional maturity of the network (Ichikawa et al., 1993).

#### 2.6.1 Network selection

Networks were selected based on the activity in each node. Up to three 2-in-1-out networks were tested simultaneously due to restrictions on the number of recording channels (approx. 280 electrodes per network, up to 1020 recording channels). Only networks that showed activity in three nodes and the connecting channel were used for the analysis. The stimulation electrode location was chosen based on previous research (Duru et al., 2023) and will be further discussed in Section 3.2.

#### 2.6.2 Data collection

Data was collected on day *in vitro* (DIV) 21 or 22. The MaxOne headstage with the chip was placed in a custom-designed incubator at 36°C, 90% humidity, and 5% CO_2_ (Duru et al., 2024). To account for temperature increases from the recording headstage during operation, the baseline temperature was set below 37°C. The recording headstage temperature remained constant throughout all experimental trials, independent of stimulation frequency or amplitude, thereby minimizing temperature-related variability in neural responses. Multiple recording headstages allowed for recording from up to four MEA chips simultaneously, meaning that on each experiment trial, up to 12 networks could be recorded. In each experiment trial, multiple stimulation paradigms were tested, resulting in continuous data collection over multiple hours.

#### 2.6.3 Stimulation and recording paradigms

Two types of experiments were performed to explore the response of the network to external stimuli: voltage amplitude-modulated experiments and frequency-modulated experiments. In both setups, biphasic pulses with a duration of 400 μs were delivered periodically to the MEA. For the amplitude-modulated experiments, the stimulation frequency was fixed at 4 Hz, as established previously (Ihle et al., 2022). The stimulation amplitude ranged from 0 mV to a maximum of 800 mV (Duru et al., 2023). In the frequency-modulated experiments, the inter-stimulus time was altered while the pulse amplitude was kept constant at 400 mV. The stimulation frequency ranged from 1 Hz to 80 Hz. Each sequence of same-amplitude or same-frequency stimuli will be further referred to as a pulse set, and each full amplitude or frequency modulation experiment will be referred to as a stimulation set. The recording of the neuronal activity was conducted simultaneously during stimulation. Output signals were recorded on electrodes in the output node (see Figures 1B iv, 2A). A time window of up to 10 ms after the input stimulus was considered as the output. For a modulation experiment, each pulse set was recorded once for a minimum of 30 seconds. The order of pulse sets was randomized. Further, it was ensured that the number of iterations used for the analysis was equivalent across all parameters within each specific experimental condition.

**Figure 2:**
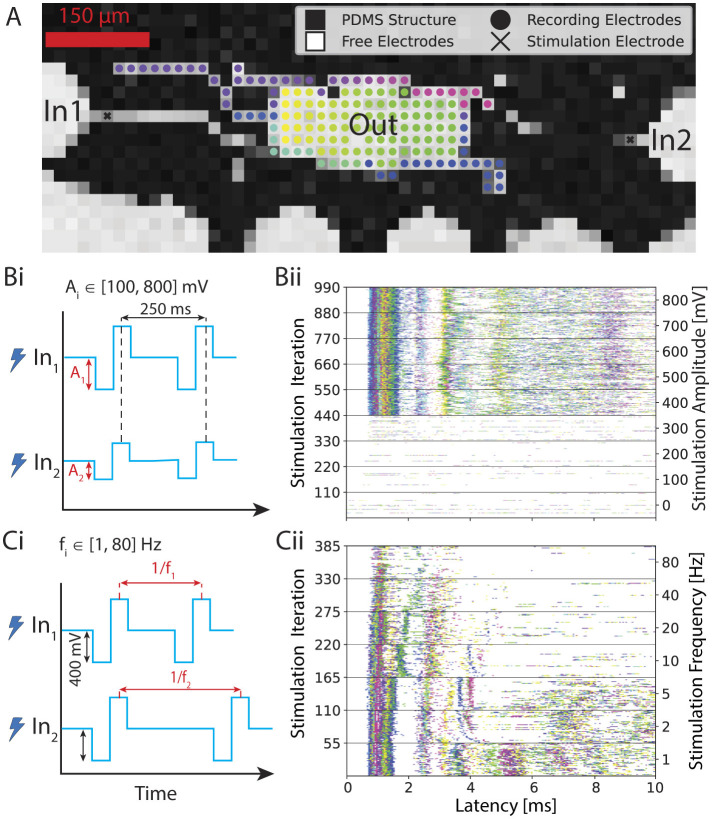
Overview of the experiment. **(A)** Voltage map showing the PDMS microstructure on a CMOS MEA. We stimulate inputs inside the axon channels and record the response from the electrodes at the output. We color-coded each recording electrode. **(Bi)** First input variant: sweeping the input voltage amplitude from 100 to 800 mV with 100 mV step size while keeping the stimulation frequency constant at 4 Hz. **(Bii)** Corresponding post-stimulus raster plots. In this case, the same stimulus is simultaneously applied 110 times for each amplitude iteration through both input electrodes. The first 10 ms of the output response is plotted after each stimulus. **(Ci)** Second input variant: sweeping through stimulation frequency from 1 to 80 Hz while keeping the stimulus voltage amplitude constant at 400 mV. **(Cii)** Corresponding post-stimulus raster plot. In this case, stimulus at the same frequency is simultaneously applied at both input electrode 1 and input electrode 2. The output response is plotted 10 ms after each stimulus.

#### 2.6.4 Data analysis

Data was processed using a custom-designed Python processing pipeline. The stimulation artifact of each recording electrode was cut out and linearly interpolated (see Supplementary Section S3 for a detailed explanation). The signal was then filtered using a second-order Butterworth high-pass filter with a cutoff frequency of 200 Hz. It was further smoothed with a 2nd-order Savitzky-Golay filter (Savitzky and Golay, 1964) using a window size of 0.25 ms to remove high-frequency fluctuations irrelevant for spike detection. Spike times were then determined using a smoothed nonlinear energy operator (SNEO) with lag *k* = 3 (Mukhopadhyay and Ray, 1998). Peaks exceeding 20 times the median of the absolute energy and no larger peak within 1.5 ms were considered spikes. The parameters for spike detection were chosen according to previous work (Maurer et al., 2024). After spike detection, an encoding method of choice (see Section 2.7.1 and Supplementary Figure S2) which maps a spike train during the response window to a scalar value was applied for each electrode. The resulting values were averaged across all recording electrodes.

### 2.7 Information encoding

In this work, four different encoding schemes (see Supplementary Figure S2) for spike trains of each experiment run were evaluated and compared in terms of performance.

#### 2.7.1 Neural Codes

Rate coding was defined as the total number of spikes within the response window. ISI encoding was also used. There, the output is interpreted by calculating the average distance between spikes. ISI is not defined if there are less than two spikes detected within the given response time window. In these cases, the ISI response was set to the duration of the considered response time window. Classical phase coding could not be used for the experiments of this work because it requires an intrinsic background oscillation of the neuronal culture as a reference (Gerstner et al., 2014). As only a short temporal segment is checked for a response, such an interpretation is not feasible. An inherited oscillatory behavior was not present in the cultures. Hence, the length of one cycle of the oscillation was defined as the time window considered. Afterward, the average phase difference of all recorded spikes was calculated. In the case of zero spikes, the phase was set to 2π − δ with δ being the sampling interval. This ensures consistency with the handling of edge cases for other temporal codes. Another encoding type that is especially used with spiking neural networks is TTFS (Eshraghian et al., 2023). There, the delay between the stimulation and the first occurring spike was measured and all remaining activity was discarded. If no spiking occurred within a given time window, TTFS was set to the length of the analyzed time window. All four encoding schemes were computed for each electrode and subsequently averaged.

#### 2.7.2 Channel capacity estimation

To assess the performance of the different encoding schemes, notions from information theory were used. A stimulus at time *t* was modeled as a discrete random variable *X*_*t*_ and the neural activity induced by the stimulus was encoded to a scalar value by one of the introduced neural codes as the discrete random variable *Y*_*t*_. The probabilistic mapping from the stimulus to *Y*_*t*_ is called a channel. It was postulated that a good encoding contains most of the information of the input stimulus. The change of the entropy *H*(·), a measure of uncertainty, of *X*_*t*_ given the response *Y*_*t*_ was investigated. This is known as the mutual information:


(1) $$\begin{array}{l} I\left(X_{t};Y_{t}\right)=H\left(X_{t}\right)-H\left(X_{t}|Y_{t}\right) \end{array}$$

On one hand, it is beneficial to effectively use a large portion of the input alphabet. On the other hand, a symbol of the alphabet must be reliably transferred to the output. This optimization problem described by the mutual information has a theoretical limit of the capacity *C*:


(2) $$\begin{array}{l} C=\underset{P\left(X_{t}\right)}{\sup}I\left(X_{t};Y_{t}\right) \end{array}$$

The capacity has the advantage over the mutual information as it does not assume any prior on *P*(*X*) and has the power to discard stimuli that only result in a highly variable output. For a discrete memoryless channel, it can be approximated with the Blahut-Arimoto (BA) algorithm (Blahut, 1972; Arimoto, 1972).

Memory was assessed by testing whether a time series *Y*_*t*|_*X*__*t*_ = *x*_ resulting from a pulse set is stationary with the augmented Dickey-Fuller test. Furthermore, significant active information storage (AIS) was evaluated with state-of-the-art estimators (Faes et al., 2011; Wibral et al., 2014; Lizier et al., 2012; Wollstadt et al., 2018). It was assumed that if there is no information gain of *Y*_*t*_ using *Y*_*t*−1_, there is also no information gain with *Y*_*t*−*i*_ ∀ *i* > 1. Based on this assumption, AIS was estimated with a lag of one by computing *I*(*Y*_*t*_; *Y*_*t*−1_|*X*_*t*_ = *x, X*_*t*−1_ = *x*) using the Kraskov estimator (Kraskov et al., 2004). The *P*-values across all stimulus parameters for both tests were combined using the weighted Stouffer's Z Method. Time series with little activity (*E*[*Y*_*t*|*X*_*t*_ = *x*_] < 0.1 with the observable *Y*_*t*|*X*_*t*_ = *x*_ being rate encoded) were given a weight of one while time series with a larger average activity had a weight of ten. AIS was non-significant (*p* > 0.05) and the time series were stationary (*p* < 0.05), supporting the conclusion that the channel can be modeled as memoryless under the tested conditions. With this memoryless assumption, *t* is left out in the subsequent part.

To have a fully discrete system, it was necessary to partition an output observation *y* ∈ ℝ into a region *b*_*i*_. For each experiment run and encoding separately, the edges of *n* bins were placed between the minimal and maximal value of the observed output *Y*. These edges were selected such that the frequency of observations was evenly distributed across all bins. An entry of the resulting transition matrix *T*, which fully describes the discrete channel, is defined as


(3) $$\begin{array}{l} T_{xi}=P\left(B=b_{i}|X=x\right). \end{array}$$

*P*(*X*) was estimated by maximizing the mutual information using *T* with the BA algorithm, which fully defines Ĥ(*X*). To approximate the conditional entropy term, the conditional distribution *P*(*Y*|*X*) was estimated in a continuous fashion using a Gaussian kernel density estimation (KDE) with a bandwidth of 2.5% of the total span of the observed values *Y*. The estimate of the channel capacity becomes


(4) $$\begin{array}{l} \hat{C}=\hat{H}\left(X\right)-\hat{H}\left(X|Y\right), \end{array}$$

with


(5) $$\begin{array}{l} \hat{H}\left(X\right)=-\underset{x}{\sum }P_{\text{BA}}\left(X=x\right)\log(P_{\text{BA}}\left(X=x\right)), \end{array}$$

and


(6) $$\begin{array}{l} \hat{H}\left(X|Y\right)=-\underset{x}{\sum }\int_{y}P_{\text{BA}}\left(x\right)P_{\text{KDE}}\left(X=x|Y=y\right)\\ \;\log(P_{\text{KDE}}\left(X=x|Y=y\right))dy. \end{array}$$

The number of bins *n* was chosen such that the bias was kept at a minimum while maintaining sufficient granularity (see Supplementary Figure S3).

## 3 Results

We report the successful long-term culturing of feedforward *in vitro* neural networks that can represent a non-linear activation function (also referred to as transfer function), *i.e*., non-linear I/O relationship. By growing these networks inside custom-designed PDMS microstructures on top of CMOS HD-MEAs (see Figure 1B i), we can stimulate the network at precise presynaptic locations. We specifically chose the electrode at the start of the input microchannel for stimulation (see Figures 1B ii, B v, 2A). This stimulation provides a means to reliably generate an action potential at the input, which then travels along the axons to the output neurons. We chose to analyze the output node as a unified/averaged response rather than examining individual neuronal activity and its interplay. We justify this approach by the high correlation observed between individual neuronal responses within nodes, as demonstrated in Supplementary Figure S11B). At our current spatiotemporal recording resolution, individual neurons within a node exhibit such similar response patterns that parsing their individual contributions would provide minimal additional information while significantly increasing analytical complexity. The high correlation suggests that neurons within a node function more as a cohesive computational unit rather than as independent processors in our specific experimental configuration. Therefore, we defined the resulting input-output relationship between the stimulated neurons in the input microchannels and the activated output neurons, which constitutes a non-linear biological transfer function.

We demonstrate that these networks can perform basic computations, such as the NAND or OR gate. In addition, we explore how the type of information encoding in neurons influences its transmission, identifying the encoding method that maximizes information transmission and efficiency.

### 3.1 Information transmission is affected by choice of neural code

**Figure 3:**
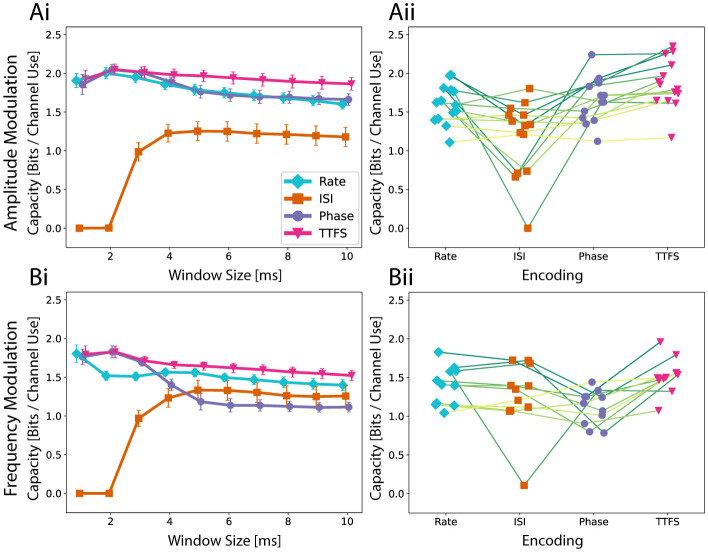
The Quality of a neural code is assessed with the estimation of the channel capacity. **(Ai)** Average capacity across fourteen networks as well as the standard error of mean for amplitude modulation. A higher capacity indicates that an encoding scheme is more expressive and reliable in transmission. The speed of the encoding is investigated by looking at multiple possible response window sizes up to 10 ms. **(Aii)** To investigate the performance differences on a network level, capacities for a response window of 10 ms are shown for each network. Encoding schemes corresponding to the same network are connected with a line. **(Bi)** Average capacity across ten networks for frequency modulation and **(Bii)** the analysis for a 10 ms time window on a network level.

Since the mechanisms of neuronal communication remain unclear, it is yet ambiguous how to interpret extracellular recordings of neurons. To choose a good method of interpreting the output signal, we compared four neural coding schemes using the concept of channel capacity. We estimated the channel capacity using the approach described in Section 2.7.2 for response window sizes of up to 10 ms. The calculated averages across multiple networks of each neural coding scheme are shown in Figure 3. We discarded networks that exhibit a smaller capacity than 1 bit per channel for any encoding from the analysis. In addition to the 10 ms response window, smaller variants were also evaluated. For both amplitude modulation (Figure 3Ai) and frequency modulation experiments (Figure 3Bi), TTFS coding outperformed the other encodings on average. As expected, most of the information is transferred during the very initial part of the response. As subsequent spikes are discarded, the noise resulting from more stochastic spiking at later stages is small, and the input can still be reliably transferred. Except for very short windows, rate coding performed slightly worse on average than TTFS coding for frequency modulation and significantly worse for amplitude modulation. There are two possible contributions to this observation. For longer windows, spikes triggered with lower probabilities are also taken into account. They lead to uncertainty in the transfer function. The occurrence of a spike for TTFS coding can alter the output value either continuously or, at a minimum, in discrete steps determined by the temporal sampling frequency of the recording system. For rate coding, such nuances are missing as the number of spikes recorded on an electrode is restricted to a small integer number due to the low cell count in the output node. This means that the accessible output range is rigid, and with limited output options, the transferred information is reduced.

Phase coding can be seen as an intermediate between TTFS and rate coding. While it does not suffer from the lack of expressiveness of rate coding, it can be more heavily affected by unreliable firing of neurons both in terms of spike occurrence and spike timing. This would explain why phase coding performed the worst for frequency modulation using a longer response window and yet was almost identical to TTFS coding when only the initial response was considered. For frequency modulation, the output inherently has a higher noise level. The capacity of ISI coding naturally behaves differently. This metric is not properly defined for small response windows as there is not enough time for multiple spikes to occur. Interestingly, ISI coding is highly robust to spiking noise occurring in later parts. While it failed to outperform any of the other neural codes for amplitude modulation, the obtained capacity for frequency modulation was comparable.

Capacity is not only determined by the encoding scheme. It also depends on inherent network properties. We observe three aspects when comparing the performances on a network level for a window size of 10 ms as seen in Figures 3Aii, Bii. Phase coding is at most as good as TTFS. This suggests that spike timings in later window parts are in general not stable enough to carry additional information as seen in the decaying capacity curve. ISI coding either performs similarly to rate coding or worse. When compared to phase coding, ISI has a similar capacity for frequency modulation but tends to score lower for amplitude modulation.

In summary, the analysis underscores the importance of the initial response window size in determining the capacity of different encoding schemes, with TTFS coding matching or outperforming others with its ability to capture nuanced timing information. Rate coding suffers from a rigid, low-resolution representation, while phase coding strikes a balance between expressiveness and susceptibility to noise. ISI coding, though inherently unsuitable for small windows, demonstrates robustness to late-stage spiking noise, particularly in frequency modulation. These findings highlight the dynamic relationship between coding strategies and network properties in maximizing information transfer. In addition, information transfer during a later part of the response is more challenging due to the inherent stochasticity of the neural network formation and behavior. However, constraining neural growth within PDMS microstructures helps mitigate complexity and reduce variability to some extent.

### 3.2 Biological neurons exhibit non-linear responses similar to activation functions used in machine learning

To investigate the transfer functions in our feedforward networks, which consist of two inputs converging into a single output, we conducted experiments using both voltage amplitude and frequency sweeps as described in Section 2.6.3. In the first experiment, biphasic rectangular voltage pulses were applied with amplitudes ranging from 100 to 800 mV in 100 mV increments (see Figure 2Bi) at a frequency of 4 Hz. Each voltage level was delivered simultaneously and identically through two electrodes located in the input axon channel (see Figure 2A), in a sequence of 120 pulses. Since a few pulses were not sent due to hardware issues and/or their response was not detected in the post-processing, we decided to keep a total of 110 pulses for the analysis to ensure an equal number of repetitions for every amplitude. Neural spikes occurring within 10 ms after each stimulus were detected and plotted to generate a post-stimulus raster plot (see Figure 2Bii), where each dot represents a neural spike on a corresponding color-coded output electrode, as defined in Figure 2A.

To minimize possible carryover effects from prior stimulation, the order of the eight same-amplitude pulse sets was randomized, and a 5-min rest period was included between stimulation sets. In Figure 2Bii, we observed a stable response per electrode, reflected in the consistent timing of spike occurrences post-stimulus. This is manifested in the observed distinct vertical “activity bands” represented in various colors depending on the electrode location. Bands with shorter latencies appear more defined and likely reflect direct action potential propagation along the input axon. In contrast, bands with longer latencies are broader and paler, indicating a lower spike frequency and greater variation in post-stimulus latency, suggesting these are likely spikes from output neurons activated via synapses between the input axon and the output neuron's dendrites. Since these spikes are the result of synaptic transmission rather than direct propagation of an elicited action potential, the stochastic component is more pronounced. They also occur less frequently, as the probability of successful synaptic transfer is less than 1, and are also less temporally precise due to expected variations in synaptic delay times (Katz and Miledi, 1965; Boudkkazi et al., 2007). It is furthermore important to note that electrodes in the middle of the output region that are located below the seeding well and therefore uncovered by the PDMS have worse signal-to-noise ratio (SNR) as opposed to the covered regions (see Supplementary Figure S4). This means that activity from neurons positioned in this region will most likely not be recorded by the system, which is visible in Supplementary Figure S4 with the whole middle region of the output not having a spike recorded within the first 5 ms upon stimulation (electrodes colored in pink).

Additionally, as can be seen in Figure 2Bii, we observed that stimulation voltages below 400 mV failed to elicit responses reliably in the output region, as indicated by the absence of activity bands. The recorded spikes are most likely due to spontaneous network activity. In contrast, voltages of 400 mV or higher consistently generated clear and regular response patterns in the output. However, the voltage amplitude at which the network reliably reacts to the applied stimulus is not necessarily identical for all networks, which will be discussed in more detail later. The observations are in line with previous work (Ihle et al., 2022; Duru et al., 2023).

**Figure 4:**
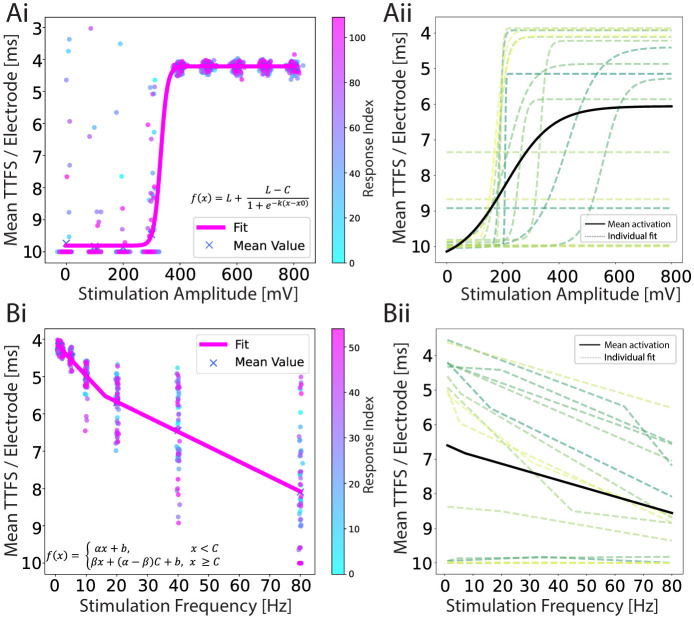
**(Ai)** An example of a response encoded as a TTFS for an amplitude-modulation experiment. A sigmoid function is fitted to a scatter plot of individual iteration responses. There are 110 responses for each amplitude and they are color-coded by time. **(Aii)** Individual sigmoid fits (dashed lines) for each network, and the mean sigmoid fit (black line) representing the average result from all experiments combined. Fit parameter values can be found in Supplementary Table S3. **(Bi)** An example of a response encoded as a TTFS for a frequency-modulation experiment. Leaky ReLU is fitted to a scatter plot of individual responses. Fifty five responses are shown for each frequency and they are color-coded by time. **(Bii)** Individual leaky ReLU fits (dashed lines) for each network, and the mean leaky ReLU fit (black line) representing the average result from all experiments combined. In both experiments, the data is recorded from 17 networks and the curves are fitted using the non-linear least squares method. Fit parameter values can be found in Supplementary Table S4. The y-axis is reverted in all plots.

For each electrode, the time of the first spike occurrence within a 10 ms latency window is taken and averaged across the electrodes in the output region, providing a mean TTFS per electrode. This encoding method is shown in Figure 4Ai, where the mean TTFS per electrode is shown as a function of stimulation voltage amplitude. The results for the remaining encodings can be found in Supplementary Figures S5 Ai–Aiii. We observe a sigmoid dependency of the TTFS on the increasing stimulation voltage with a broad response distribution for amplitudes at or above 400 mV. In Figure 4Aii, we present sigmoid fits for various individual 2-in-1-out networks (dashed lines) and the fit calculated over all collected data points for the experiment in question (solid line). While networks show reproducibility in the overall behavior, they display variability in saturation values and around the transition region, which is attributable to several factors.

The heterogeneity of neuronal networks introduces inherent variability in the input and output cluster characteristics. Due to the stochastic nature of cell seeding and potential cell loss during culture, cell cluster sizes differ, ranging from single cells to populations containing tens of cells (see Supplementary Figure S6). These spatial distribution and inter-connectivity differences can substantially influence neuronal activation patterns, particularly manifesting in variations of the sigmoid response curve's plateau values. Additionally, axonal excitability is spatially dependent, with proximity to stimulation electrodes directly influencing action potential initiation. Networks with closer axonal adherence require lower stimulation voltages to trigger action potentials (Grosberg et al., 2017; Fernandes et al., 2023), resulting in a variable input range for a transition region.

The overall sigmoid profile can be influenced by the choice of the stimulation electrode. In our approach, we recorded activity in the input channel region for 30 s and selected the electrode with the highest activity as the stimulation electrode. This criterion was intended to ensure that stimulation targeted an active axon, however, high activity upstream does not necessarily correlate with robust response elicitation downstream in the network.

In the second set of experiments, we applied a biphasic stimulation pulse at a fixed voltage amplitude but varied the frequency from 1 to 80 Hz, as shown in Figure 2Ci. We picked 400 mV for the stimulation amplitude because this was the first amplitude above the threshold that we identified with the previous experiment. Seven frequencies were chosen: 1, 2, 5, 10, 20, 40, and 80 Hz. The number of iterations in the same time window for each frequency was in this case variable because it directly depends on the frequency. To ensure a fair comparison between the different parameters, the 1 Hz frequency pulse set was applied for twice the duration. For frequencies exceeding 2 Hz, a subset of responses was selected to maintain an equal number of samples for each parameter. Additionally, the time intervals between retained samples were standardized and maximized and only responses that resulted from stimulation on both input electrodes were kept. An example of a post-stimulus raster plot during this frequency sweep is presented in Figure 2Cii. We observed higher output activity at lower stimulation frequencies (1–5 Hz), with a marked decrease in induced activity at higher frequencies. Additionally, the activity bands within the first 2 ms were more stable at lower frequencies.

These results suggest that high-frequency stimulation leads to activity depletion, a phenomenon previously noted in studies of deep brain stimulation at 130 Hz (Yousif et al., 2012). The frequencies that deplete activity (80–185 Hz) (Garcia et al., 2003; Griffin et al., 2011) align with the higher frequencies used in our experiments. Other studies have demonstrated partial or complete blockage of neural activity, depending on the relationship between the stimulation frequency and the neuron's intrinsic firing frequency (Ye et al., 2022).

The corresponding TTFS is given in Figure 4Bi and shows an increase with increasing stimulation frequency. A leaky rectified linear unit (ReLU) is fitted to the data and shown in pink. The results for the remaining encodings can be found in Supplementary Figures S5Bi–Biii. In Figure 4Bii we show the mean leaky ReLU of the 15 different 2-in-1-out networks. It is visible that for some networks stimulation evoked negligible activity (there were no spikes recorded within the 10 ms window on average), but for most networks, we observed the characteristic curve when encoding the output response in the form of TTFS.

In general, stimulation of 2-in-1-out neural networks shows reproducible behavior for both stimulation paradigms when stimuli with different amplitude or frequency are applied simultaneously at both input locations. Furthermore, amplitude and frequency sweep provide two distinct activation functions. The encoded output response is noisy due to intrinsic network activity as well as inevitable variations of the experimental parameters. For both input types, the response index shows a uniform distribution of response values over time (see Figures 4Ai, Bi), which means that we do not induce long-term modification in the network behavior with our stimulation paradigms. The insensitivity of the observed response to the randomization of the applied amplitude and frequency sequence also supports this claim.

### 3.3 Fundamental logic operations with biological neurons

**Figure 5:**
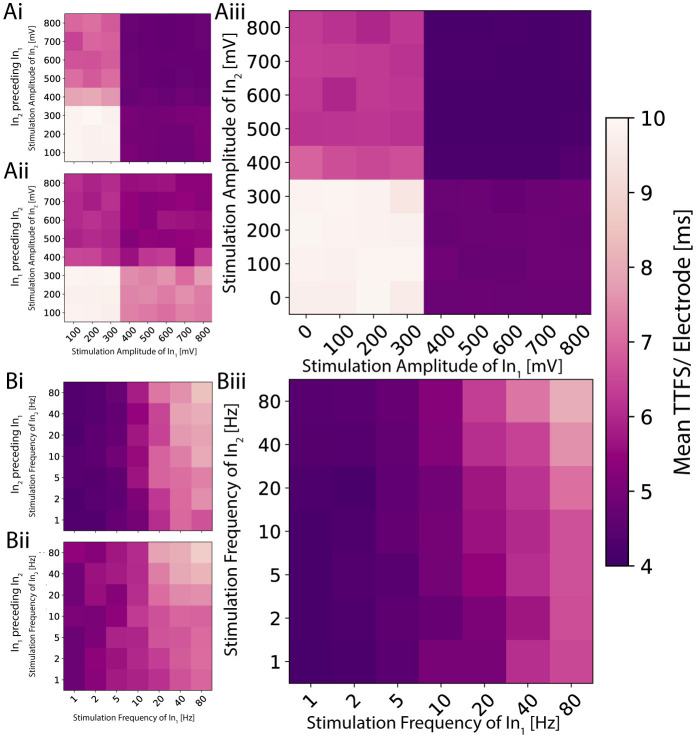
2D planes illustrating the activation function for distinct signals on the two inputs for a single network. **(Ai)** The amplitude is modulated on the two inputs independently, and the stimulation pulse sent to the second input precedes the one on the first by 1.2 ms. **(Aii)** The order is reversed. **(Aiii)** Both pulses are sent simultaneously. The resulting activation resembles a logic AND or OR gate depending on the applied threshold. **(Bi)** The frequency is modulated, and the periodic signal sent to the second input is shifted 1.2 ms earlier in time. **(Bii)** The shift is reversed. **(Biii)** Both signals are synchronized. The resulting activation exhibits low-pass behavior, analogous to a logic NOR or NAND gate depending on the used threshold.

In this section, we expand on the findings of Section 3.2 and now simultaneously apply either distinct voltage amplitudes or different frequencies to the chosen stimulation electrodes in the input channels. Additionally, we introduce an optional delay of 1.2 ms between the pulses of one iteration to investigate its effect on activation patterns (see Supplementary Figure S7). The latter pulse is considered the start of the response to mitigate potential issues of stimulation artifacts. The resulting activation patterns are illustrated in Figure 5.

As previously seen in Figure 4Ai, voltages above a certain threshold elicited a slightly noisy yet consistent response for amplitude modulation when stimulating both electrodes with the same amplitude simultaneously. In this experiment, we investigated if and how the measured TTFS depends on which of the two pulses exceeds the threshold. We found four distinct regions within the activation function with high TTFS values in the bottom left quadrant, low values in the top right, and intermediate values in the remaining two quadrants (see Figure 5A). Analogies can be drawn to the operation of fundamental logic gates. In logic gates, multiple input bits are combined through logic operations to produce a single output bit. Similarly, if one maps the observed TTFS and the given input amplitudes to bits through thresholding, the network exhibits behavior akin to OR or AND gates. All tested neural codes showed an equivalent behavior as seen in Supplementary Figure S8. By applying a delay to one of the inputs, it is possible to shift the relative weighting between the two stimuli. The contribution of the earlier applied stimulus decreases as seen in Figures 5Ai, Aii. This is in agreement with the observation seen in Figure 2Bii that the initial response during the first few milliseconds gives a strong and stable band and becomes more sparse for later time points.

Frequency modulation exhibits a less evident pattern (see Figure 5B) compared to amplitude modulation. The TTFS encoding scheme shows a continuous low-pass characteristic in the mean response. While this trend remains observable, it is less pronounced for the other three neural codes (Supplementary Figure S8). As previously illustrated in Figure 4Bii, frequency modulation indeed provides a more nuanced and smoother nonlinear output response. However, these responses also exhibit significant variability at higher frequencies. Combined with the capacity analysis presented in Figure 3B—which indicates an upper limit of approximately 1.5 bits per channel use—it becomes challenging to reliably encode more than two distinct states. Nevertheless, stable responses observed at low frequencies, along with distinctly different average delays at higher frequencies, enable the mapping of bits to frequencies in a manner suitable for implementing logic NOR or NAND operations. Similar observations hold true for delayed versions of the input stimulus, mirroring those made for amplitude modulation, with the delayed input stimulus typically becoming more dominant.

This behavior is consistent across different networks. For both modulations, one of the two stimuli has a larger impact compared to its counterpart. For the amplitude modulation, this means that the more influential stimulus gives rise to earlier spikes across the whole output region. For frequency modulation in general, high stimulation frequency results in low activity in the output. The input that has a weaker response for amplitude modulation will have severely less impact on the output for frequency modulation. This suggests that an input connection is responsible for a specific sub-region of the output. Overstimulated neurons of the first input node will no longer elicit a response in the neurons of the output node which can only be partially compensated by the neurons of the second input node. This would allow for more targeted control of a network's output.

## 4 Discussion

This study contributes to our understanding of how small biological neuronal networks with controlled topology process information, suggesting that their input-output relationships can be characterized as non-linear activation functions. Our findings indicate that these minimal networks can execute fundamental operations resembling Boolean logic, such as NAND and OR, through simple two-input, one-output architectures. The relatively consistent responses observed across different preparations represent a promising step toward addressing one of the key requirements we identified for effective biohybrid computation: defining basic computational capabilities of biological neural systems. Furthermore, our analysis highlights the role of output encoding schemes in determining the quantity and quality of information transmitted through the system. Our work identifies certain limitations of rate coding for information transmission due to its reliance on temporal averaging, which can result in information loss. Among the alternative spike-time output encoding schemes examined (TTFS, ISI, and phase coding), only TTFS performed better or on par with rate coding. This observation raises important questions about whether timing information is not being captured adequately in our experimental paradigm, or whether rate coding—despite its simplicity—can capture substantial information content, a point also discussed by others (Brette, 2015). Brette argues that the rate vs. timing debate is less about which coding scheme is fundamentally relevant, but rather about whether rate-based approaches can adequately capture neural behavior. Our findings suggest that selecting an appropriate coding scheme should be guided by the specific computational objectives of the biocomputing system.

In this work, we tried to leverage the potential of a high-density recording readout and its spatio-temporal precision while capturing the important characteristics of the network dynamics. While each individual neuron pair in our system could be treated as an individual non-linear operator, the choice had to be made to balance the system in terms of functionality, robustness, efficiency, and experimental possibilities. Treating neural activity as population activity in this case offered several advantages. For one, it accounted for noise. Individual neurons exhibit stochastic firing patterns and considerable variability in their responses. By averaging across a population, random fluctuations tend to cancel out, revealing underlying signal patterns more clearly. Furthermore, it is speculated that information in neural systems is often encoded across multiple neurons rather than in single cells (Georgopoulos et al., 1986; Averbeck et al., 2006). Population activity captures these distributed representations that might be missed when focusing on individual neurons. Then, as mentioned earlier, neurons within local circuits often work as functional units with highly correlated activity patterns. Therefore, examining individual neurons adds analytical complexity without providing proportional additional insights. On the other hand, precisely controlled networks with structured, non-random connectivity at the single-neuron level would likely yield valuable additional insights—a direction we are actively pursuing in our upcoming work and touched upon in our past work regarding the experimental realization of it (Mateus et al., 2022; Duru et al., 2023; Vulić et al., 2024).

While our work provides insights into the fundamental processing capabilities of simplified biological networks, it represents only an initial step toward the broader goal of harnessing biological computation. The remaining challenges include developing methods to integrate these basic computational units into more complex hierarchical systems and gaining precise control over their communication pathways—specifically the number, strength, and directionality of synaptic connections. For instance, classical logic circuits composed of NAND gates can theoretically implement arbitrary computations, such as half- or full-adders. However, directly emulating such circuits in biological neuronal networks might be inefficient, potentially requiring extensive interconnections and a substantial number of neuronal units as it does not try to exploit the inherent properties of biological neurons. Additionally, reliably interconnecting these neuronal units into functional hierarchies presents significant difficulties, particularly regarding suitable encoding strategies at both input and output stages. An intuitive approach might involve using frequency modulation as the input encoding while interpreting outputs through a form of rate coding, though the identification of an optimal scheme remains an open question. Alternatively, introducing a distinct encoding specifically for inter-network communication could simplify integration. Another possible approach involves artificially mediating interconnections between networks, translating between input and output encodings externally; however, this would shift part of the computational load away from the biological neurons themselves, which would not be necessarily desirable, especially in the context of computational efficiency.

There are long ways to go yet to fully realize the potential of biocomputing. To progress beyond basic operations, several advancements are necessary. A crucial next development would be incorporating learning mechanisms through stimulation paradigms that enable short-term and long-term modulation of synaptic weights. This could shift our approach from passively characterizing inherent network behavior to actively shaping computational capabilities for specific problem-solving. The ability to modify biological synaptic weights would allow direct examination of learning rules commonly implemented in artificial bio-inspired systems (Serb et al., 2020). Additionally, enabling real-time online interaction would enhance control and adaptability of these biological systems (Voitiuk et al., 2024; Maurer et al., 2024). Further exploration of input encoding strategies is also needed to optimize information transmission. To improve understanding and reduce variability, as mentioned earlier, simplifying network topology to study single-neuron I/O relationships could prove valuable. Much of the variability in our current neural systems stems from inadequate control over network subpopulations and synaptic connectivity within and between neural nodes. Previous studies have demonstrated how network topology influences information processing in spiking neural networks (Downes et al., 2012; Dimovska et al., 2019), highlighting the importance of addressing these methodological challenges. Achieving comprehensive control and systematically correlating topology with computational characteristics will be essential for advancing hybrid intelligence and biocomputing systems. The adaptability of PDMS design platforms offers a promising experimental approach to empirically validate whether *in silico* proposed network topologies can translate effectively into functional biological neural architectures.

To summarize, understanding how biological networks process information at a fundamental level enhances the plausibility and fidelity of bio-inspired AI systems. Incorporating these principles into AI models can improve their generalization, learning efficiency, and adaptability, aligning them more closely with human cognitive capabilities. The ability to execute Boolean logic is a foundational requirement for computation. If small biological networks can perform these tasks, it validates the potential of spiking and bio-inspired systems to handle more complex logical and algorithmic operations at scale. This study attempts to bridge the gap between biological insights and computational implementation, facilitating advancements in energy-efficient, scalable, and biologically plausible AI systems.

## Data Availability

The raw data supporting the conclusions of this article is available at ETH Research Collection: https://doi.org/10.3929/ethz-b-000729744. The code used for data analysis is available at https://github.com/Hullimulli/engineered_biological_neural_networks_as_basic_logic_operators.

## References

[B1] Abramson C. I. (1990). Invertebrate Learning: A Laboratory Manual and Source Book. Washington, DC: American Psychological Association. 10.1037/10078-000

[B2] Adu K. Yu Y. Cai J. Asare I. Quahin J. (2021). The influence of the activation function in a capsule network for brain tumor type classification. Int. J. Imaging Syst. Technol. 32, 123–143. 10.1002/ima.22638

[B3] Amos G. Ihle S. J. Clément B. F. Duru J. Girardin S. Maurer B.. (2024). Engineering an *in vitro* retinothalamic nerve model. Front. Neurosci. 18:1396966. 10.3389/fnins.2024.1396966 38835836 PMC11148348

[B4] Appeltant L. Soriano M. C. Van der Sande G. Danckaert J. Massar S. Dambre J.. (2011). Information processing using a single dynamical node as complex system. Nat. Commun. 2:468. 10.1038/ncomms1476 21915110 PMC3195233

[B5] Arimoto S. (1972). An algorithm for computing the capacity of arbitrary discrete memoryless channels. IEEE Trans. Inf. Theory 18, 14–20. 10.1109/TIT.1972.1054753

[B6] Averbeck B. B. Latham P. E. Pouget A. (2006). Neural correlations, population coding and computation. Nat. Rev. Neurosci. 7, 358–366. 10.1038/nrn1888 16760916

[B7] Bakkum D. J. Frey U. Radivojevic M. Russell T. L. Müller J. Fiscella M.. (2013). Tracking axonal action potential propagation on a high-density microelectrode array across hundreds of sites. Nat. Commun. 4:2181. 10.1038/ncomms3181 23867868 PMC5419423

[B8] Beniaguev D. Segev I. London M. (2021). Single cortical neurons as deep artificial neural networks. Neuron 109, 2727–2739.e3. 10.1016/j.neuron.2021.07.002 34380016

[B9] Blahut R. (1972). Computation of channel capacity and rate-distortion functions. IEEE Trans. Inf. Theory 18, 460–473. 10.1109/TIT.1972.1054855

[B10] Boudkkazi S. Carlier E. Ankri N. Caillard O. Giraud P. Fronzaroli-Molinieres L.. (2007). Release-dependent variations in synaptic latency: a putative code for short- and long-term synaptic dynamics. Neuron 56, 1048–1058. 10.1016/j.neuron.2007.10.037 18093526

[B11] Brette R. (2015). Philosophy of the spike: rate-based vs. spike-based theories of the brain. Front. Syst. Neurosci. 9:140675. 10.3389/fnsys.2015.00151 26617496 PMC4639701

[B12] Brewer G. J. Torricelli J. R. Evege E. K. Price P. J. (1993). Optimized survival of hippocampal neurons in B27-supplemented neurobasal™, a new serum-free medium combination. J. Neurosci. Res. 35, 567–576. 10.1002/jnr.490350513 8377226

[B13] Burr G. W. Shelby R. M. Sebastian A. Kim S. Kim S. Sidler S.. (2016). Neuromorphic computing using non-volatile memory. Adv. Phys. X 2, 89–124. 10.1080/23746149.2016.1259585

[B14] Cazé R. D. Humphries M. Gutkin B. (2013). Passive dendrites enable single neurons to compute linearly non-separable functions. PLoS Comput. Biol. 9:e1002867. 10.1371/journal.pcbi.1002867 23468600 PMC3585427

[B15] Chicca E. Stefanini F. Bartolozzi C. Indiveri G. (2014). Neuromorphic electronic circuits for building autonomous cognitive systems. Proc. IEEE 102, 1367–1388. 10.1109/JPROC.2014.2313954

[B16] Courte J. Renault R. Jan A. Viovy J. L. Peyrin J. M. Villard C. (2018). Reconstruction of directed neuronal networks in a microfluidic device with asymmetric microchannels. Methods Cell Biol. 148, 71–95. 10.1016/bs.mcb.2018.07.002 30473075

[B17] De Venuto G. Beaubois R. Zahedi S. Caré M. Cheslet J. Barban F.. (2024). “Recapitulating the electrophysiological features of in vivo biological networks by using a real-time hardware spiking neural network,” in 2024 46th Annual International Conference of the IEEE Engineering in Medicine and Biology Society (EMBC) (IEEE), 1–4. 10.1109/EMBC53108.2024.10781591 40039599

[B18] Dimovska M. Johnston T. Schuman C. D. Mitchell J. P. Potok T. E. (2019). “Multi-objective optimization for size and resilience of spiking neural networks,” in 2019 IEEE 10th Annual Ubiquitous Computing, Electronics &Mobile Communication Conference (UEMCON), 0433–0439. 10.1109/UEMCON47517.2019.8992983

[B19] Downes J. Hammond M. W. Xydas D. Spencer M. Becerra V. M. Warwick K.. (2012). Emergence of a small-world functional network in cultured neurons. PLoS Comput. Biol. 8:e1002522. 10.1371/journal.pcbi.1002522 22615555 PMC3355061

[B20] Duru J. Küchler J. Ihle S. J. Forró C. Bernardi A. Girardin S.. (2022). Engineered biological neural networks on high density CMOS microelectrode arrays. Front. Neurosci. 16:829884. 10.3389/fnins.2022.829884 35264928 PMC8900719

[B21] Duru J. Maurer B. Giles Doran C. Jelitto R. Küchler J. Ihle S. J.. (2023). Investigation of the input-output relationship of engineered neural networks using high-density microelectrode arrays. Biosens. Bioelectr. 239:115591. 10.1016/j.bios.2023.115591 37634421

[B22] Duru J. Maurer B. Ruff T. Vulić K. Hengsteler J. Girardin S.. (2024). A modular and flexible open source cell incubator system for mobile and stationary use. HardwareX 20:e00571. 10.1016/j.ohx.2024.e00571 39678521 PMC11639333

[B23] Eshraghian J. K. Ward M. Neftci E. O. Wang X. Lenz G. Dwivedi G.. (2023). Training spiking neural networks using lessons from deep learning. Proc. IEEE. 111, 1016–1054. 10.1109/JPROC.2023.3308088

[B24] Faes L. Nollo G. Porta A. (2011). Information-based detection of nonlinear granger causality in multivariate processes via a nonuniform embedding technique. Phys. Rev. E 83:051112. 10.1103/PhysRevE.83.051112 21728495

[B25] Fahimirad M. Kotamjani S. S. (2018). A review on application of artificial intelligence in teaching and learning in educational contexts. Int. J. Learn. Dev. 8:106. 10.5296/ijld.v8i4.1405736467167

[B26] Fernandes S. R. Pereira M. Elbasiouny S. M. Dhaher Y. Y. de Carvalho M. Miranda P. C. (2023). Interplay Between Electrical Conductivity of Tissues and Position of Electrodes in Transcutaneous Spinal Direct Current Stimulation (tsDCS). Cham: Springer International Publishing, 101–122. 10.1007/978-3-031-15451-5_7

[B27] Forró C. Thompson-Steckel G. Weaver S. Weydert S. Ihle S. Dermutz H.. (2018). Modular microstructure design to build neuronal networks of defined functional connectivity. Biosens. Bioelectr. 122, 75–87. 10.1016/j.bios.2018.08.075 30243047

[B28] Garcia L. Audin J. D'Alessandro G. Bioulac B. Hammond C. (2003). Dual effect of high-frequency stimulation on subthalamic neuron activity. J. Neurosci. 23, 8743–8751. 10.1523/JNEUROSCI.23-25-08743.2003 14507974 PMC6740410

[B29] Garliauskas A. (2007). Information conveyed by the neural network systems and its applied significance. Informatica 18, 203–216. 10.15388/Informatica.2007.172

[B30] Georgopoulos A. P. Schwartz A. B. Kettner R. E. (1986). Neuronal population coding of movement direction. Science 233, 1416–1419. 10.1126/science.3749885 3749885

[B31] Gerstner W. Kistler W. M. Naud R. Paninski L. (2014). Neuronal Dynamics: From Single Neurons to Networks and Models of Cognition. Cambridge, UK: Cambridge University Press. 10.1017/CBO9781107447615

[B32] Griffin D. M. Hudson H. M. Belhaj-Saïf A. Cheney P. D. (2011). Hijacking cortical motor output with repetitive microstimulation. J. Neurosci. 31, 13088–13096. 10.1523/JNEUROSCI.6322-10.2011 21917792 PMC3187611

[B33] Grosberg L. E. Ganesan K. Goetz G. A. Madugula S. S. Bhaskhar N. Fan V.. (2017). Activation of ganglion cells and axon bundles using epiretinal electrical stimulation. J. Neurophysiol. 118, 1457–1471. 10.1152/jn.00750.2016 28566464 PMC5596129

[B34] Guo W. Fouda M. E. Eltawil A. M. Salama K. N. (2021). Neural coding in spiking neural networks: a comparative study for robust neuromorphic systems. Front. Neurosci. 15:638474. 10.3389/fnins.2021.638474 33746705 PMC7970006

[B35] Hamming R. W. (1990). The Art of Probability for Scientists and Engineers. London, UK: Longman Higher Education.

[B36] Headley D. B. Paré D. (2017). Common oscillatory mechanisms across multiple memory systems. NPJ Sci. Learn. 2:1. 10.1038/s41539-016-0001-2 30294452 PMC6171763

[B37] Hodgkin A. L. Huxley A. F. (1952). A quantitative description of membrane current and its application to conduction and excitation in nerve. J. Physiol. 117, 500–544. 10.1113/jphysiol.1952.sp004764 12991237 PMC1392413

[B38] Ichikawa M. Muramoto K. Kobayashi K. Kawahara M. Kuroda Y. (1993). Formation and maturation of synapses in primary cultures of rat cerebral cortical cells: an electron microscopic study. Neurosci. Res. 16, 95–103. 10.1016/0168-0102(93)90076-3 8387174

[B39] Ihle S. J. Girardin S. Felder T. Ruff T. Hengsteler J. Duru J.. (2022). An experimental paradigm to investigate stimulation dependent activity in topologically constrained neuronal networks. Biosens. Bioelectr. 201:113896. 10.1016/j.bios.2021.113896 35032845

[B40] Ikeda S. Manton J. H. (2009). “Spiking neuron channel,” in 2009 IEEE International Symposium on Information Theory. 10.1109/ISIT.2009.5205817

[B41] Indiveri G. Linares-Barranco B. Hamilton T. J. Schaik A. v. Etienne-Cummings R. Delbruck T.. (2011). Neuromorphic silicon neuron circuits. Front. Neurosci. 5:73. 10.3389/fnins.2011.00073 21747754 PMC3130465

[B42] Jaeger H. Haas H. (2004). Harnessing nonlinearity: Predicting chaotic systems and saving energy in wireless communication. Science 304, 78–80. 10.1126/science.1091277 15064413

[B43] Kagan B. J. Kitchen A. C. Tran N. T. Habibollahi F. Khajehnejad M. Parker B. J.. (2022). *in vitro* neurons learn and exhibit sentience when embodied in a simulated game-world. Neuron 110, 3952–3969.e8. 10.1016/j.neuron.2022.09.001 36228614 PMC9747182

[B44] Karki S. Chavez Arana D. Sornborger A. Caravelli F. (2024). Neuromorphic on-chip reservoir computing with spiking neural network architectures. arXiv preprint arXiv:2407.20547.

[B45] Katz B. Miledi R. (1965). The measurement of synaptic delay, and the time course of acetylcholine release at the neuromuscular junction. Proc. R. Soc. London 161, 483–495. 10.1098/rspb.1965.0016 14278409

[B46] Kayser C. Montemurro M. A. Logothetis N. K. Panzeri S. (2009). Spike-phase coding boosts and stabilizes information carried by spatial and temporal spike patterns. Neuron 61, 597–608. 10.1016/j.neuron.2009.01.008 19249279

[B47] Kim M. Lee J. (2019). Ferroelectric analog synaptic transistors. Nano Lett. 19, 2044–2050. 10.1021/acs.nanolett.9b00180 30698976

[B48] Kraskov A. Stögbauer H. Grassberger P. (2004). Estimating mutual information. Phys. Rev. E 69:066138. 10.1103/PhysRevE.69.066138 15244698

[B49] Kuebler E. Thivierge J. (2014). Spiking variability: theory, measures and implementation in matlab. Quantit. Methods Psychol. 10, 131–142. 10.20982/tqmp.10.2.p131

[B50] Kwisthout J. Donselaar N. (2020). “On the computational power and complexity of spiking neural networks,” in Proceedings of the Neuro-Inspired Computational Elements Workshop, 1–7. 10.1145/3381755.3381760

[B51] Lampl I. Yarom Y. (1997). Subthreshold oscillations and resonant behavior: two manifestations of the same mechanism. Neuroscience 78, 325–341. 10.1016/S0306-4522(96)00588-X 9145790

[B52] Lavi A. Sehgal M. Sisan F. Okabe A. Silva A. J. (2021). A retrograde mechanism coordinates memory allocation across brain regions. bioRxiv. 10.1101/2021.10.28.46636136563678

[B53] Lee C. Sarwar S. S. Panda P. Srinivasan G. Roy K. (2020). Enabling spike-based backpropagation for training deep neural network architectures. Front. Neurosci. 14:119. 10.3389/fnins.2020.00119 32180697 PMC7059737

[B54] Lee I.-K. Kwon S. (2010). Design of sigmoid activation functions for fuzzy cognitive maps via Lyapunov stability analysis. IEICE Trans. Inf. Syst. E93, 2883–2886. 10.1587/transinf.E93.D.288312739966

[B55] Lizier J. T. Prokopenko M. Zomaya A. Y. (2012). Local measures of information storage in complex distributed computation. Inf. Sci. 208, 39–54. 10.1016/j.ins.2012.04.016

[B56] London M. Roth A. Beeren L. Häusser M. Latham P. E. (2010). Sensitivity to perturbations in vivo implies high noise and suggests rate coding in cortex. Nature 466, 123–127. 10.1038/nature09086 20596024 PMC2898896

[B57] Lundstrom B. N. Fairhall A. L. (2006). Decoding stimulus variance from a distributional neural code of interspike intervals. J. Neurosci. 26, 9030–9037. 10.1523/JNEUROSCI.0225-06.2006 16943561 PMC6675329

[B58] Maass W. (1997). Networks of spiking neurons: the third generation of neural network models. Neural Netw. 10, 1659–1671. 10.1016/S0893-6080(97)00011-7

[B59] Maoz B. M. (2021). Brain-on-a-chip: Characterizing the next generation of advanced *in vitro* platforms for modeling the central nervous system. APL Bioeng. 5:030902. 10.1063/5.0055812 34368601 PMC8325567

[B60] Mateus J. C. Weaver S. Van Swaay D. Renz A. F. Hengsteler J. Aguiar P.. (2022). Nanoscale patterning of *in vitro* neuronal circuits. ACS Nano 16, 5731–5742. 10.1021/acsnano.1c10750 35404570

[B61] Maurer B. Fassbind S. Ruff T. Duru J. Spacone G. Rodde T.. (2024). Inkube: An all-in-one solution for neuron culturing, electrophysiology, and fluidic exchange. bioRxiv. 10.1101/2024.12.06.627248 PMC12951282 41766565

[B62] Mead C. (1990). Neuromorphic electronic systems. Proc. IEEE 78, 1629–1636. 10.1109/5.58356

[B63] Mukhopadhyay S. Ray G. C. (1998). A new interpretation of nonlinear energy operator and its efficacy in spike detection. IEEE Trans. Biomed. Eng. 45, 180–187. 10.1109/10.661266 9473841

[B64] Park S. Kim S. Choe H. Yoon S. (2019). “Fast and efficient information transmission with burst spikes in deep spiking neural networks,” in Proceedings of the 56th Annual Design Automation Conference 2019, DAC '19, New York, NY, USA (Association for Computing Machinery). 10.1145/3316781.3317822

[B65] Rathi N. Roy K. (2023). Diet-SNN: a low-latency spiking neural network with direct input encoding and leakage and threshold optimization. IEEE Trans. Neural Netw. Learn. Syst. 34, 3174–3182. 10.1109/TNNLS.2021.3111897 34596559

[B66] Roy D. S. Kitamura T. Okuyama T. Ogawa S. Sun C. Obata Y.. (2017). Distinct neural circuits for the formation and retrieval of episodic memories. Cell 170, 1000–1012.e19. 10.1016/j.cell.2017.07.013 28823555 PMC5586038

[B67] Savitzky A. Golay M. J. (1964). Smoothing and differentiation of data by simplified least squares procedures. Anal. Chem. 36, 1627–1639. 10.1021/ac60214a04721322220

[B68] Seo K. Tang J. Roll I. Fels S. Yoon D. (2021). The impact of artificial intelligence on learner-instructor interaction in online learning. Int. J. Educ. Technol. High. Educ. 18:2. 10.1186/s41239-021-00292-9 34778540 PMC8545464

[B69] Serb A. Corna A. George R. Khiat A. Rocchi F. Reato M.. (2020). Memristive synapses connect brain and silicon spiking neurons. Sci. Rep. 10:2590. 10.1038/s41598-020-58831-9 32098971 PMC7042282

[B70] Shahaf G. Marom S. (2001). Learning in networks of cortical neurons. J. Neurosci. 21, 8782–8788. 10.1523/JNEUROSCI.21-22-08782.2001 11698590 PMC6762268

[B71] Sidiropoulou K. Pissadaki E. K. Poirazi P. (2006). Inside the brain of a neuron. EMBO Rep. 7, 886–892. 10.1038/sj.embor.7400789 16953202 PMC1559659

[B72] Smirnova L. Caffo B. S. Gracias D. H. Huang Q. Morales Pantoja I. E. Tang B.. (2023). Organoid intelligence (oi): the new frontier in biocomputing and intelligence-in-a-dish. Front. Sci. 1:1017235. 10.3389/fsci.2023.1017235

[B73] Tanaka G. Yamane T. Héroux J. Nakane R. Kanazawa N. Takeda S.. (2019). Recent advances in physical reservoir computing: a review. Neural Netw. 115, 100–123. 10.1016/j.neunet.2019.03.005 30981085

[B74] Ünal H. T. Ba s cift ci F. (2023). Neural logic circuits: an evolutionary neural architecture that can learn and generalize. Knowl. Based Syst. 265:110379. 10.1016/j.knosys.2023.110379

[B75] Voitiuk K. Seiler S. T. de Melo M. P. Geng J. Hernandez S. Schweiger H. E.. (2024). A feedback-driven iot microfluidic, electrophysiology, and imaging platform for brain organoid studies. bioRxiv.38559212

[B76] Vulić K. Amos G. Ruff T. Kasm R. Ihle S. J. Küchler J.. (2024). Impact of microchannel width on axons for brain-on-chip applications. Lab Chip 24, 5155–5166. 10.1039/D4LC00440J 39440578 PMC11497309

[B77] Wibral M. Lizier J. T. Vögler S. Priesemann V. Galuske R. (2014). Local active information storage as a tool to understand distributed neural information processing. Front. Neuroinform. 8:1. 10.3389/fninf.2014.00001 24501593 PMC3904075

[B78] Wollstadt P. Lizier J. T. Vicente R. Finn C. Martinez-Zarzuela M. Mediano P.. (2018). IDTxl: The Information Dynamics Toolkit xl: a Python package for the efficient analysis of multivariate information dynamics in networks. arXiv [Preprint]. arXiv:1807.10459.

[B79] Ye H. Chen V. Hendee J. (2022). Cellular mechanisms underlying state-dependent neural inhibition with magnetic stimulation. Sci. Rep. 12:12131. 10.1038/s41598-022-16494-8 35840656 PMC9287388

[B80] Yousif N. Borisyuk R. Pavese N. Nandi D. Bain P. (2012). Spatiotemporal visualization of deep brain stimulation-induced effects in the subthalamic nucleus. Eur. J. Neurosci. 36, 2252–2259. 10.1111/j.1460-9568.2012.08086.x 22805069

[B81] Yu Y. Adu K. Tashi N. Anokye P. Wang X. Ayidzoe M. A. (2020). RMAF: relu-memristor-like activation function for deep learning. IEEE Access 8, 72727–72741. 10.1109/ACCESS.2020.2987829

[B82] Zador A. Claiborne B. Brown T. (1991). “Nonlinear pattern separation in single hippocampal neurons with active dendritic membrane,” in Advances in Neural Information Processing Systems, eds. Moody J. Hanson S. Lippmann R. (Morgan-Kaufmann).

[B83] Zeng Y. Ferdous Z. I. Zhang W. Xu M. Yu A. Patel D.. (2019). Inference with hybrid bio-hardware neural networks. arXiv 1905.11594.

